# Topic: Returns to education in Ghana: The role of education of one's father

**DOI:** 10.1016/j.heliyon.2024.e37805

**Published:** 2024-09-11

**Authors:** Emmanuel AduBoahen, Paul Hammond, Paul A. Kwakwa

**Affiliations:** University of Energy & Natural Resources, Department of Entrepreneurship and Business Sciences, P. O. Box 214, Sunyani, Ghana

**Keywords:** Education, Instrumental variable, Regression, Wages, Ghana

## Abstract

The study examines the role of education of father on returns to schooling in Ghana. The data used for the analysis is the Ghana Living Standards Survey Seven (GLSS 7). Spouse education is used as an instrument in our estimation. Father's education, other demographic characteristics and employment characteristics were controlled in the study. Regression analysis revealed that returns on education in Ghana are positively affected by fathers' level of education. It was found that respondents whose fathers have no education were found to obtain lower wages than their counterparts whose fathers had higher levels of educational certification. The evidence was consistent across the formal and informal sectors as well as private and public sector workers. Given that Ghanaians in wage employment are small, the benefits of the study would have been much greater if non wage employees were included in the analysis. The study recommends policies that would enhance educational attainment but reduce parental influence in job acquisition. Furthermore, Policies to formalise the labour market of Ghana will further enhance the returns to schooling in Ghana.

## 1 Introduction

It is commonly acknowledged that education is a vital human right and a key engine for economic and social transformation [1]. Education is one of the ways through which a country can achieve upwards social mobility and poverty eradication. As such, it plays a crucial role in driving both economic and social development. The return on education refers to the economic gains that people or communities can realise by investing in education. This can be assessed by comparing the average lifetime earnings of those with advanced degrees of education to those with lower levels of certificates [2]. Additionally, the return on education can be determined by means of its broader social and economic advantages, such as higher economic growth and decreased poverty [3]. Several studies have examined the relationship between education and income over the years [4,5]. However, the connection between education and income is not always straightforward. To actually ascertain whether the observed relationship between education and income is causal is very challenging, as there are unobserved factors that affect both education and income; hence, omitting such variables may lead to a biased estimate.

Empirical research has demonstrated the link between education and earnings as well as the variables affecting the return on investment in education. The return on education varies by educational degree, the field of study, and other individual attributes, according to several studies, and it is positively correlated with income and job options [4,6]. However, according to other research, the connection between education and income is more nuanced and may be influenced by factors such as job qualities, institutional considerations, and market frictions [4,7].

One area that has not received much attention is the influence of the educational level of one's parents in determining the educational attainment and earning potential of an individual [4]. Within the context of the relationship between education and the return on education, the transmission of education from one generation to the next is an essential component. Some studies have concluded that an individual's educational attainment and wages are positively correlated with the individual's parents' level of education [4,8]. It is argued that those who have parents with better levels of education have a greater chance of achieving higher levels of education themselves, and as a result, they have a greater possibility of earning more money. This correlation can be partially explained by the fact that parents who have completed higher levels of education have a greater likelihood of having higher salaries, which allows them to give their children superior educational chances and resources. Another possible explanation is that the father's educational level may be a better indicator of the family's socioeconomic background and the resources available to the family. Additionally, the father's education level may influence the values and expectations that children develop regarding education and career aspirations [9,10].

Surprisingly, several empirical studies that aim to ascertain the causal impact of education on wages often fail to control for the education of one's parents [10]. In this study, we use the education of one's father instead of the education of a parent that has the highest education. This is because Ghana is a patriarchal country; therefore, men tend to possess much power in the household, and they often make many decisions in the household. The implicit assumption in most of these studies failing to control for the education of the father is that their instrumental variable is most likely not to be correlated with the education of one's father. If this implicit assumption is true, then their estimates may not be biased, but if it fails, then their estimate may be biased. Most at times, earlier researchers rely on various variables on family background as instruments, but such instruments are likely to correlate with both fathers' education and earnings due to intergenerational effects. However, these earlier studies used family background, such as expenditure on education, spouse education, sibling education, etc., as an instrument of education have failed to control for fathers' education in their model. These family background characteristics are most likely to correlate with the education of one's father, and therefore, failing to control for one's father's education may lead to an inconsistent estimate of returns to schooling.

In many developing countries, income levels are low. However, Ghana's income level is lower than that of many other developing countries. This is partly attributed to lower wages and salaries. Thus, aside from the fact that GDP is low, issues including lower labour productivity are mentioned to be behind low income levels in Ghana [11]. Over the years, there has been back and forth debate between employers and workers about increasing wages to trigger higher productivity or vice versa. That notwithstanding, the belief that education can trigger higher productivity to fetch workers higher remuneration has been identified by government and policy makers in Ghana. This is evidenced by certain educational policies that have been implemented. For instance, the early 1990s saw implementation of the FCUBE policy aimed at increasing basic school enrolment. Records show that this policy supported higher enrolment and completion rates [12]. Since 2017, free senior high school education running with similar objectives has been operational. Tertiary education has expanded through the increased number of universities, colleges of education and nursing colleges. Despite all these, it appears that the country is yet to witness wages associated with educational levels [13]. How does education affect wage returns in Ghana?

In an attempt to address the research question, it is important to consider the issues raised in the earlier paragraphs. It is observed in the literature that parental education, especially that of fathers, correlates positively with educational status and even job opportunities of their children [14]. Thus, our primary objective is to investigate the importance of fathers' education towards the returns to schooling of an individual. The study also set out to perform heterogeneity analysis by investigating the role of fathers’ education in public‒private and formal-informal sectors of the Ghanaian economy.

Our article adds to the nascent literature on returns on education in developing countries, especially among sub-Sahara African countries. Our contribution, relative to the existing studies, takes into consideration an important variable such as father's education, which when omitted from the returns to education model, would likely bias the regression estimates. The second contribution of our paper to the literature is demonstrating that the returns on education in Ghana are not uniform across various sectors of the economy. Given that the returns to education may change over time, using more recent data for such analysis may help in policy making. However, recent studies on the returns to education have only concentrated on urban Ghana, and those studies that study the returns to schooling for the entire country were done more than 20 years ago. Thus, our study provides a new piece of evidence on returns to schooling using a more recent national representative dataset to conduct the analysis.

The remainder of the paper is organised as follows: Section 2 presents a review of the relevant literature. Section 3 provides a brief background on education and the labour market in Ghana. In section 4, we discuss the econometric framework used in our analysis. Section 5 presents data and provides a descriptive analysis of the data. The results are presented in section 6. Section 7 provides a robustness check. A discussion of the findings is presented in section 8, while the last section concludes the paper.

## 2 Literature review

This literature review seeks to provide both theoretical and empirical synthesis of the research that has been conducted over the years regarding the relationship between education and earnings. We will provide a review of four theories that underpin the returns to schooling: human capital theory, signalling theory, social closure theory and matching theory.

### 2.1 Theoretical literature on returns on education

Human capital theory is a paradigm that explains how individuals can boost their earning potential and contribute to the greater economy by acquiring marketable skills and information through education. According to this theory, education and training equip individuals with valuable skills and information that may be used to boost workplace efficiency, which then leads to higher earnings and economic expansion [15]. Proponents of this theory often argue that financial investments in education and training are comparable to those made in tangible assets such as machinery and equipment [16]. Both investment types boost output and support economic expansion. Other arguments by human capital theorists are that the value of soft skills such as cooperation and communication at the workplace are essential for efficient production and that education and training are one of the media that aid people in developing these talents [17]. The theory has been criticised for its narrow view on returns to individual investments in education and training by neglecting important cultural and institutional variables that may also contribute to returns to education. For example, discrimination and job market segmentation might reduce the economic returns of schooling for some populations.

While human capital theory provides a useful framework for understanding the relationship between education and earnings, signalling theory offers an alternative explanation. Education, rather than being an outright investment in human capital, is seen as a signal sent to prospective employers, in accordance with signalling theory [18]. The concept of education as a signal to potential employers of one's skills and personality traits is explained by the widely used framework of signalling theory. Education, according to the theory, can be a pricey but worthwhile signal to prospective employers of a candidate's intelligence, work ethic, and persistence. The assumption is that people who already possess these traits are more likely to make an effort to further their education, allowing them to set themselves apart from those who lack them [19]. emphasised that education can serve as a signal of an individual's unobserved abilities, such as intelligence, persistence and motivation, which cannot be directly observed by employers. A major critique of signalling theory is that it may lead to "credential inflation," in which companies may require applicants to have more education than it is actually needed for the post.

In the context of labour markets, social‐closure theory on returns to education points to institutional regulations that define requirements and thresholds for job entry. Limiting access to particular occupations creates distortions in the labour market, which increase the earnings of individuals employed in the restricted industry [20,21]. Since excluding incompetent people from job appointments is important, employers need to find the best ways to exclude these incompetent individuals. Using individuals' educational backgrounds has become a more socially acceptable means of excluding people as incompetent rather than using one's unconscious biases toward racism or sexism or excluding other factors not related to competence [20].

The final theoretical framework for comprehending the connection between education and income is provided by matching theory. The theory is used to explain how job matching works in the labour market. The fundamental idea is that job seekers and employers must look for one another in a decentralised market. Information asymmetries, job preferences, and job characteristics all play a role in how matches are made. This theory states that people with higher education levels are more likely to find higher-paying jobs that call for specialised skills [22]. Job matching models must take into account the heterogeneity of job seekers and employers and the frictions and search costs that arise in the process of search and matching [23,24]. The effectiveness of job matching in the labour market, for instance, can be increased by measures that eliminate information asymmetries, such as job search help programmes or job fairs. Additionally, by expanding the pool of competent job candidates and minimising skill mismatches, policies that support education and training can enhance the quality of job matching.

### 2.2 Empirical review of return to education

Much research has been conducted on how education affects the earnings of an individual. Here, we look at the results of several studies on the return to education with much emphasis on Ghana [4]. used data from household surveys to determine the private returns to education in 48 African countries. They found that the returns to education were generally positive and significant, with tertiary education showing the highest returns. However, they also said that the returns were different from country to country and that access to education and the benefits of education were different for men and women. Similarly [25], found the returns to education in SSA to be 12.4 %, which is higher than the world average of 9.7 %. Their findings lend support to those theoretical arguments that educated people in Africa can give better signals to employers and are more likely to be matched to better jobs, thereby making them have higher earnings.

In a recent study [10], found that the return on education is higher in jobs with more complex tasks and more freedom. In another study [26], found that the return on education is higher for jobs that require more skills and have a wider range of tasks. Their findings suggest that education gives people skills that are more useful in these types of jobs. Hence, their empirical study supports the human capital theory of returns to schooling. In the United States of America, some studies have investigated why the wage gap between white and black Americans is narrowing, and they found improvement in the quality of education of black schools contributing to the reduction [27,28]. In Brazil [29], found that omitting family background from the returns to education model may lead to overestimation of the returns to schooling. Findings from a recent study in China indicate that the returns to schooling in the formal sector of China are significantly higher for graduates from high school and university than for graduates from the informal sector [30]. The authors found that the returns to schooling of university graduates are greater than those of high school graduates in both sectors.

In Ghana, there are several studies on returns to schooling. For example, a study by Ref. [31] argued that the returns to schooling in the country are between 5.4 and 6.0 percent. Using propensity score matching to study the returns to education in Ghana [32], found that the returns to schooling for primary school leavers are not different from those who did not attend primary school. However, they found the rate of return for middle school and secondary school graduates to be between 82.9 and 86.1 % for nongovernment wage earners and 43.6–44.4 % for self-employed. Similar studies have found the returns to schooling in Ghana to be increasing with an increasing level of schooling [[33], [34], [35]]. In a similar study [36], found that the income of nonfarm self-employees increased by 4.1 percent for any additional year of schooling. He further found that any additional year in schooling of a family member with the highest education in the household increases the profit of an individual living in the household by 3.3 percent. Other studies in the country that try to disaggregate the returns to schooling by gender have found the returns to education of women to be slightly higher than those of men [33,35]. Several of these earlier studies in the country failed to account for omitted variable such as ability, which affect both education and earnings. Studies that attempt to address the issue of omitted variable bias (OVB) and are therefore close to our study are [33], which uses spouse education as an instrument, and [34], which uses family background characteristics to address the possibility of omitted variable bias in the earnings equation. However, using family background characteristics as instrument is questionable, as it is most likely to correlate with education and earnings and therefore may not satisfy the exclusion restriction assumption of an IV. However [33], failed to control for father's education in his model, but father's education is likely to correlate with one's spouse's education and earnings. In fact, omitting father's education when spouse education is used as an instrument for education may still cause regression results to be biased attempt to address that. Again, earlier studies in the country have not considered the contribution of father's education towards the returns to schooling, and the study aims to fill that literature gap.

## 3 Brief background to the labour market and educational system in Ghana

### 3.1 Ghanaian educational system

Despite recent progress in school enrolment and improved levels of educational attainment, Ghana's education system continues to face significant challenges, including poor infrastructure, insufficient funding, and a shortage of trained teachers. Notwithstanding these challenges, educating Ghanaian children is seen by policy makers and other important stakeholders as a sure way to improve the country's human capital, which would eventually lead to economic development and improvement in welfare.

Education in Ghana ranges from preschool to tertiary. Children below the age of 6 years often attend preschool, which often comprises two years of Nursery and one year of kindergarten. The official age to begin primary 1 in Ghana is six years, but this official age of entering primary 1 is not enforced in the country. In view of that, there can be early entry and late entry into primary 1. The structure of Ghanaian education can broadly be categorised into three categories, namely, basic education, secondary education and tertiary education. Basic education comprises 6 years of primary education and 3 years of junior high school. This level of schooling is offered by both private and public schools. To a large extent, private schools operating at this level are seen to perform better than public schools. Thus, most average Ghanaian households prefer to send their children to private basic schools rather than public basic schools. Children from wealthy homes often attend private basic schools even though attending public basic school is virtually free. At the end of the 9 years of basic school education, the country conducts a nationwide examination for students at this level, and those who passed this examination use the grade they obtained in the examination to access the next level of education.

The next level of education after basic school is often referred to as senior high school (SHS) or senior secondary school (SSS), which is equivalent to high schools in other countries. This level of schooling requires 3 years. At this level, a few students opt for Technical Vocational Education and Training (TVET), while the majority of students opt for general education. There are very few private schools operating at this level. In contrast to basic schools, public schools at this level perform better than private schools. Schools at this level are categorised into classes A, B, C, and D. For one to obtain access to Class A schools, that individual should obtain a high grade in the nationwide examination organised at the basic level. Because children from private basic schools often perform better than their counterparts in public schools, they are the very children who often get access to these Class A schools, and such children often come from wealthy homes likely to be headed by educated parents. At the end of the 3 years of schooling at this level, a nationwide examination is organised for students at this level to be used to enter tertiary school.

The tertiary level of education in Ghana consists of polytechnic/technical universities, training colleges and university education. Training colleges and polytechnics requires either a minimum of 2 or 3 years to complete, while university education requires a minimum of 4 years. Largely, training colleges and polytechnics award diploma certificates, and universities award bachelors or postgraduate certificates. There are some few instances where some universities train some of their students for two years and award them Diploma. Compared to private tertiary schools, public tertiary schools perform better than their private counterparts. Public tertiary institutions in the country do not pay tuition fees, making them more affordable than private tertiary institutions. Several of the students who found themselves in private tertiary institutions either failed to get their programme of choice in public tertiary schools or did not get admission to public tertiary schools due to the limited number of spaces in public tertiary schools.

### 3.2 Labour market

Ghana has a population of approximately 31 million people and is located in West Africa. The country's labour market is characterised by high levels of informal employment, underemployment, and a mismatch between labour force skills and economic needs. In terms of employment, the majority of Ghanaians work in the informal sector, which accounts for more than 80 % of total employment [37]. Low productivity, low wages, and limited access to social protection characterise the informal sector. In contrast, the formal sector, which includes industries such as mining, manufacturing, and services, offers higher wages and better working conditions but is only available to a small percentage of the labour force.

The inability of the labour force in Ghana to possess the skills that are required by the economy is one of the most significant obstacles that the Ghanaian labour market must overcome. It has been argued that the educational system in Ghana does not effectively prepare students for the requirements of the labour market. High rates of youth unemployment and underemployment can be attributed to a lack of training and education that is sector-specific as well as lacking in practical skills.

In recent years, the government of Ghana has undertaken efforts to address these concerns through reforms to the education system and initiatives aimed at fostering employment and skill development. These reforms and initiatives are examples of how the government is attempting to solve the issues. For instance, the government has initiated a variety of vocational and technical training programmes in an effort to provide students with marketable skills and to prepare them for professions in a variety of different fields [38]. In addition, the government has started programmes such as the National Youth Employment Program, which gives young people options for jobs and training at the same time.

Despite all of these efforts, Ghana's education system and its labour market continue to face severe obstacles. The high levels of informality and underemployment in the labour market limit chances for economic growth and social mobility. At the same time, the inadequate funding of the school system and poor infrastructure continue to impair the quality of education needed to feed the labour market.

## 4 Econometric framework

The study analyses the returns to education of persons in wage employment and then examines how the failure to control for education of a father may lead to omitted variable bias. The study first presents estimations of returns on education without considering two important issues: (1) the fact that education might be endogenous since one's ability can affect both years of schooling and wages and (2) the likelihood that people may self-select themselves into wage employment. We address the endogeneity problem by using instrumental variable estimations and the self-selection problem by using the Heckman selection procedure. Finally, we empirically show how the returns on education may vary depending on the level of education of one's father.

To efficiently estimate the coefficient of schooling in equation (1), two important econometric issues needs to be addressed. First, schooling in our model suppose to be exogenous—meaning that there should be no variable in the error term in equation (1) that correlates with schooling. However, schooling in equation (1) is unlikely to be exogenous. For example an individual's ability is likely to correlate with years of schooling as well as wages. This suggests that schooling in equation (1) is most likely to be endogenous. To address the endogeneity problem that may lead to a bias estimate for schooling, the study adopted an Instrumental Variable (IV) approach. The approach is to find a variable that is likely to correlate with schooling but can affect wages only through schooling (dubbed ‘exclusion restriction’). Spouse education is likely to have a positive correlation with one's education and also satisfy the exclusion restriction assumption [33,39]. Thus, spouse education is used as an instrument in the study. Second, there is the possibility of self selection into the labour market. It is expected that people with high schooling are more likely to participate in the labour market. If that is the case, then the coefficient of equation (1) would be biased if we fail to address the self-selection problem [40]. correction procedure has widely been used to address this self-selection problem and this study follows this correction procedure to address the possibility of self-selection problem in our model.

The empirical model often adopted to measure private returns on education is the Mincerian earning function (see, inter alia, [33,41]. The equation is given as:

(1) $$\ln\;w_{i}=\alpha +\delta S_{i}+\beta X_{i}+\varepsilon_{i}^{1}$$

Where $\ln\;w_{i}$ is the monthly log wage of individual *i*, $S_{i}$ is the total years of schooling of employee *i*, and $X_{i}$ is a vector of personal characteristics likely to affect the wages of individual *i*. Following the work of [33], we adopted similar explanatory variables. The explanatory variables used in our wage equation include age and the square of age as proxies to capture experience and experience squared, respectively. Our choice of age as proxy for experience is the lack of information on experience in the dataset we are using for our analysis. Other personal characteristics, such as urbanicity of residency, type of industry, household size, household head, permanent employment, religion and ethnicity, are also used as explanatory variables, as they are likely to affect wages. $\varepsilon_{i}$ is the idiosyncratic error term, which is assumed not to contain any other variable that affects both wages and years of education.

In Ghana, businesses often prefer to set up in areas with improved infrastructures. This suggests that urbanicity of residency is likely to affect wages and the probability of being engaged in wage employment. Both religion and ethnicity are likely to affect wage employment as belonging to a religious or ethnic group can create social networks than can enhance one's employment opportunities. On the contrary, belonging to some religious group may have a negative effect of one's employment opportunities as some employers may see some religious believes to have a negative effective on productivity. Even though, some temporal employment such consultancy services often offer higher wages than many of the permanent employment, several of the temporal employments in the country often offer lower wages than permanent employment. Thus, there is a higher likelihood that being in permanent employment will increase wages. Certain industries provide higher remuneration than others and it is therefore necessary to control for industry type. For example, individuals employed in the agric sector often receive lower wages than those employed in the manufacturing sector.

In the situation whereby $\varepsilon_{i}$ is uncorrelated with $S_{i}$, we can estimate equation (1) by OLS. However, several studies have demonstrated that an individual's ability, which is unobserved, may likely affect both years of education and wages (see, inter alia, [[42], [43], [44]]. In such a situation, $\varepsilon_{i}$ will correlate with $S_{i}$, and therefore, estimating equation (1) with OLS would lead to a biased estimate. To address this endogeneity problem, instrumental variable (IV) estimation has been suggested in many empirical studies [33,41,45,46]. Following [39], our study adopts the years of schooling of a spouse as an instrument of total years of education. Spouse education is likely to correlate with one's education and is likely to satisfy the exclusion restriction assumption of an IV.

The use of IV to address the problem in equation (1) calls for a first-stage regression estimate for years of education, which can be represented in equation (2) as follows:

(2) $$S_{i}=\vartheta +{\tau SP}_{i}+\omega X_{i}+u_{i}$$

where ${SP}_{i}$ is spouse years of schooling, $S_{i}$ and $X_{i}$ have the same definition as in equation (1) and $u_{i}$ is the idiosyncratic error term.

In most labour markets that do not exclude the Ghanaian labour market, there is often self-selection in participation in the labour force. In most instances, individuals' participation in the labour market, especially women, is affected by other factors, such as marriage and childcare [47,48]. Heckman's two-step procedure is used to correct the nonrandomness in participation in the labour force as shown in equation (3) below.

The correction of sample selection using Heckman's two-step procedure involves first estimating the equation:

(3) $$prob\left(L_{i}=1\right)={a+rT}_{i}+v_{i}$$

where $L_{i}$ is the labour force participation of individual *i*, $T_{i}$ represents the vector of explanatory variables and $\nu_{i}$ is the idiosyncratic error term, which is assumed to have a conditional mean of zero [40]. has shown that under setting assumptions, equation (1) can be represented as

(4) $$\ln\;w_{i}=\alpha +\delta S_{i}+\beta X_{i}+c\gamma_{i}+\varepsilon_{i}^{2}$$

where $\gamma_{i}=\frac{\phi \left(\mu P_{i}\right)}{\Phi \left(\mu \psi \right)}$ and ϕ,Φ are the standard normal density and the normal distribution, respectively. If we assume that $\varepsilon_{i}^{2}$ has a conditional mean of zero, δ can consistently be estimated if $S_{i}$ is appropriately instrumented using equation (2). The inverse Mills ratio (IMR) is identified by the exclusion restriction assumption. Following earlier studies, we used an indicator variable of a child below 6 years in the household, an indicator variable of an individual above 60 years in the household, an indicator variable of marriage and household size as exclusion restrictions [45,46,48].

Having a child in the household may affect participation in the labour market as childcare is time intensive but ones an individual participate in the labour market and get a wage employment, having a child in the household may not affect the wages of the individual. Similar to having a child in the household, having an older person above 60 years may likewise have a negative effect on labour market participation but not on wages. On the contrary, having an older person above 60 years can also increase labour market participation of an adult in the household as the older person can take care of the children in the households making time for the adult in the household to participate in the labour market. In Ghana, marriage often imposes a burden on women as most customs of several of the ethnic groupings in the country demands married women to perform all the household chores and taking care of the children in the household. This means that marriage may affect labour market participation especially for women but it highly unlikely that marriage my affect wages. From the forgoing argument, the four variables; an indicator of a child below 6 years old, an indicator variable of an individual above 60 years in the household, an indicator variable of marriage and household size satisfies the [40] exclusion restriction assumption.

Several empirical studies on returns to schooling attempt to address the above endogeneities to estimate the causal effect of private return to schooling. The major challenge confronting empirical researchers is to find a suitable instrument that is correlated with schooling but uncorrelated with the explained variations in wages. Most at times, earlier researchers rely on various variables on family background as instruments, but such instruments are likely to correlate with earnings due to intergenerational effects. Recently, the cost of schooling, distance to schooling, and educational reforms that affect years of schooling have been used as instruments. The major issue is that some of these instruments require administrative data, which are rare to obtain in developing economies, and some of the educational reforms come with economic reforms, which therefore may correlate with wages.

[33,39] used spouse education as an instrument of years of education in Ghana. Although this instrument is likely to satisfy the exclusion restriction assumption, the instrument is also likely to be endogenous, as spouse education is likely to partially correlate with one's father's education. However, the earlier study in Ghana that used spouse education as an instrument to study the returns to education failed to control for the education of the father, which makes it difficult to justify that the conditional expectation of the error term in equation (4) is zero. In fact, using spouse education without controlling for father's education may have been a perfect instrument for years of education if the labour market in Ghana functions well such that parental social capital does not affect the time and type of job a person gets after graduating from school. However, that is not the case for Ghana and many developing countries. The study uses the education of the father as a proxy for parental social capital. In an imperfect labour market such as Ghana and that of most developing countries where parental social capital has a strong influence on obtaining work in highly paid jobs, omitting such an important variable may also lead to an inconsistent estimate for the returns to schooling when the instrument used for years of education correlates with parental social capital. Father's education is a good proxy for parental social capital, as friendship is often built by school mates and at workplaces, thus making educated fathers well connected.

Some earlier studies have even used fathers' education as an IV for years of education [14,45,46] which indicates that fathers' education is likely to partially correlate with children's education. However, father's education as an instrument may be questionable due to possibilities of intergenerational transfer effects. The study is therefore in support of spouse education as an IV but only argues that father's education ought to be controlled before a consistent estimate can be obtained. We therefore modify equation (4) to include father's education so that our regression equation would now be formulated as follows:

(5) $$\ln\;w_{i}=\alpha +\delta S_{i}+\beta X_{i}+hF_{i}+c\gamma_{i}+\varepsilon_{i}^{3}$$

Where $F_{i}$ is the education of one's father. Estimating equation (5) by using spouse education for $S_{i}$ will lead to a consistent estimate for δ. The statistical software used for all the estimations is Stata 14.

## 5 Data and descriptive statistics

This study uses information from the latest nationally representative household survey in Ghana, dubbed the Ghana Living Standards Survey Seven (GLSS 7). The dataset adopted a two-stage stratified sampling design to collect information on individuals. In the first stage, 1000 enumerated areas were selected to form primary sampling units. The list of the enumerated areas for which the samples were drawn was obtained from the 2010 Population and Housing Census and these primary sample units were allocated into the 10 administrative regions in the country using probability proportional to population size. A complete listing of all the households in the selected primary sampling units were undertaken to form the secondary sampling units. In the second stage, 15 households were selected systematically from each of the primary sampling units. A total of 15,000 households were selected across the country, of which 14,009 were successfully interviewed. A total of 31,305 people of working age (15–60 years) were interviewed. Similar to many other studies, we excluded disabled individuals from the sample. Due to problems associated with measuring the earnings of unpaid entrepreneurs or employees, the primary focus of the study would be on wage workers. Second, the dataset we are using does not provide information on the earnings of nonwage workers.

The dataset for the study is restricted between ages 15 and 60 years. We excluded individuals above age 60 years just to avoid retirees since the official age for retirement in Ghana for most formal wage workers is 60 years.^1^ To avoid censoring people's years of schooling, we excluded those who were still in school at the time of the survey. Excluding children, old age, disabled^2^ and those still in school leaves us with a total sample size of 22,123 out of which 4533 are in wage employment, 14,400 are in nonwage employment and the remaining are out of the labor force. We retrieve information on personal characteristics such as literacy, education, age, marital status, religion, ethnicity, household size, number of children in a household, urbanicity of residence, wages, labour market participation, hours worked in a week, type of work and the type of industry one's occupation belongs.

The study defines education as the total years of schooling which is estimated as the highest level of education attained prior to the survey. Both no education and pre-primary education is counted as zero. Thus, the counting of the years of schooling begins from primary 1. Repetition is not considered in the counting of the years of schooling. For example, if a person in Primary 3 has repeated twice before reaching that stage, the years of schooling of that individual is considered as 3 years even though the person might have spent 5 years at that stage. In this study, marriage is defined as an adult male and female who come together under the customary, religious or legal marital laws of the country or two consenting adults of the opposite sex who are cohabiting. A household consists of a person or a group of persons, who live together in the same house or compound, share the same house-keeping arrangements and are catered for as one unit. The head of household is that individual who has the economic and social responsibility of the household. Both household and household head are retrieved directly from the dataset. An individual in our study is considered literate if that individual can read or write basic English language and solve some basic arithmetic questions that were administered by the enumerators. All individuals who received monetary rewards for their employment are classified as wage employees. Labour market participation refers to adults who are gainfully employed or having being actively searching for job 7 days prior to the survey.

Father's level of education is divided into five categories depending on the highest level of certification of an individual's father. The categories include (1) no certificate—the education of an individual's father is below the minimum requirement for one to receive the Basic Education Certificate Examination (B.E.C.E)/JSS certificate, (2) Basic certificate—individual have the B.E.C.E/JSS certificate, (3) secondary certificate—the individual completed secondary/high school and took part in the nationwide secondary school examination of which students are awarded the Senior Secondary School Certificate Examination, (4) Diploma certificate and (5) Degree Certificate or higher. There are several religion in Ghana but the major ones are Christian and Islam. The study therefore divided religion into 3 categories; Christian, Islam and Other religion. Where other religion includes all forms belief system aside Christianity and Islam.

In the dataset, responses from respondents concerning the duration of receiving wages from their employer were in different time units. To have a uniform measure on the time units of receiving wages, we converted all the different units of receiving wages to monthly wages. From the dataset, 69.7 percent of the wage earners reported that they receive their payment monthly, 19.7 percent reported that they receive their payment daily, and 9.2 percent said they receive their payment weekly. The remaining 1.4 percent reported in different time units, such as biweekly and quarterly. The study defines marriage as individuals who are either customary or legally married or two individuals from the opposite sex who are cohabiting. Literacy used in this article refers to those individuals who could read and write some basic tests in English and solve some basic math problems that were administered to them during the survey.

Table 1 provides descriptive statistics of the sampled data used for this study. There are three columns in Table 1: the first column presents the characteristics of people in wage employment, the second column presents the characteristics of those in nonwage employment, and the final column presents the characteristics of those who are out of the labour force. Those who are out of the labour force are likely to be younger compared to their counterparts in any form of employment. The table further reveals that those who are out of the labour force are less likely to marry and be a household head but more likely to live in a larger household. It is not surprising that people in wage employment live in a smaller household and have less likelihood of living in a household that has a child below five years or an adult above 60 years. The lower flexibility in wage employment actually forces people in wage employment to reduce their dependency in terms of caring for children and the aged. On the other hand, people with more of such dependency may opt to stay out of the labour force or engage in nonwage employment, such as self-employment, which is more flexible. The table suggests that Christianity is the dominant religion in the country and that the Akan tribe is the dominant ethnic group in the country. For example, as high as 77 percent of those in wage employment are Christians, and the number slightly decreases to 65 and 66 percent for nonwage employment and out of labor force, respectively. Those in wage employment are more likely to have other members in the household who are wage workers than those in nonwage employment or those who are out of the labor force.

**Table 1:** Descriptive statistics.

	Wage employment	Non-Wage Employment	Out of labour force
	(1)	(2)	(3)
*Demographics*			
Age in years (Mean)	34.58	36.22	29.74
Married	0.47	0.57	0.34
Household size (Mean)	4.32	5.96	6.05
Household head	0.64	0.42	0.22
1 if Children below 5 in hh	0.45	0.57	0.47
1 if Aged (60+) in hh	0.13	0.21	0.27
Christian	0.77	0.65	0.66
Islam	0.16	0.23	0.24
Other religion	0.07	0.19	0.10
Akan tribe	0.48	0.35	0.36
Mole tribe	0.18	0.27	0.33
Ewe tribe	0.14	0.11	0.07
Other tribes	0.20	0.27	0.23
1 if other wage employee in the hh	0.29	0.02	0.05
*Education*			
Literacy	0.62	0.30	0.35
Years of schooling (Mean)	10.57	5.01	5.20
*Location*			
1 if urban	0.64	0.33	0.43
*Education of Father and Spouse*			
Spouse years of schooling	7.10	4.12	4.25
Father's years of schooling	6.27	3.27	4.06
1 if father has no certificate	0.50	0.73	0.70
1 if father has JSS certificate	0.38	0.23	0.23
1 if father has SSS certificate	0.06	0.02	0.03
1 if father has Diploma certificate	0.03	0.01	0.02
1 if father has Graduate or above	0.03	0.01	0.01
*Employment related variable*			
Hourly worked (mean)	34.83	0.51	–
Monthly wage	748.66.68	–	–
1 if permanent	0.76	0.68	–
N	4533	14,400	3190

*Note:*hh is household. Children below 15 years of age and adults above 60 years of age were excluded. Individuals who are still in school and the disabled are excluded. Religion include 3 categories namely; Christian, Islam and other religion; tribe include 4 categories namely; Akan, Ewe, Mole and other tribe; Father's certificate have five categories, namely; no certificate, JSS certificate, SSS certificate, Diploma Certificate and Graduate or a higher certificate.

Table 1 further reveals that those in wage employment are more literate, have more years of schooling and are more likely to live in urban communities in Ghana. For example, the average years of schooling for wage employees are more than twice those of their counterparts who are not in wage employment. Years of schooling of fathers and spouses of people in wage employment are higher than their counterparts in nonwage employment or out of the labor force. The table reveals the mean hourly work for wage employees to be 34.83 h in a week compared to 0.51 h by nonwage workers. Wage employees are most likely to be in permanent employment compared to those in nonwage employment. Given that the dollar-to-cedi exchange rate at the time of the survey is 4.6, the average monthly wage converted into dollars is 162.75.

Table 2 provides employment characteristics across different levels of education. The table reveals an interesting pattern between the level of education and employment. The table shows that people with basic education are more likely to be employed in agriculture or the trading industry but are less likely to work as professionals. For example, as low as approximately 12 % of people without a certificate work as professionals, but having at least a secondary school certificate increases the figure to almost six times. In terms of hours of work, there is not much difference across levels of education. It can be seen from the table that the proportion of people working as permanent employees increases steadily along the various levels of education—from 70 % to approximately 95 %. This suggests that those with higher educational certificates tend to have more job security than their counterparts with lower educational certificates. In Ghana, apart from some casual or part-time employment, such as consultancy work, which seeks to attract better remuneration and conditions of service, casual or part-time employment tends to pay lower wages than permanent employment. It can be seen from the table that there are higher returns on education. For example, the wages of people with a degree or higher are approximately five times their counterparts with a basic certificate or lower and more than double those holding a diploma certificate.

**Table 2:** Employment characteristics across different levels of certification.

	Highest Certificate				
	No certificate	Basic Certificate	Secondary Certificate	Diploma Certificate	Degree or higher
1 if Agriculture	17.67	6.12	2.07	3.30	5.38
1 if Professionals	12.08	37.24	70.70	74.50	75.00
1 if sellers	10.49	12.75	2.71	3.85	1.79
1 if Manufacturing	7.84	7.24	3.18	2.39	4.46
Hours work per week	34.51	36.89	32.53	34.74	36.81
Wages in Ghana Cedis	586.91	547.72	926.80	1228.55	2601.96
1 if Permanent wage employee	70.01	72.65	89.81	91.19	95.54

Note: Individuals below 28 years and adults above 60 years are excluded. Disabled individuals are excluded. The figures for the dichotomous variables are in percentages.

In Table 3, we present the results of the same employment variables as in Table 2 but this time across different levels of certificates of one's father. Table 3 exhibits a trend similar to that of Table 2 except that the margins of the percentage changes are smaller for that of Table 3. For example, whilst the wages of people who have their father's holding degree or higher are approximately twice their counterparts who have their fathers having a basic certificate or lesser, it is rather five times when we are rather comparing wages across educational levels of individuals but not their fathers. Similarly, we observe wages of people with their fathers having degrees or higher to be higher than their counterparts with fathers holding diploma by only 20 % but it is more than 100 % when we are actually doing the comparison across people educational levels rather than their father.

**Table 3:** Employment characteristics across different levels of father's educational certification.

	Highest Certificate of Father				
	No certificate	Basic Certificate	Secondary Certificate	Diploma Certificate	Degree or higher
1 if Agriculture	14.66	9.11	5.60	4.61	1.83
1 if Professionals	29.42	32.98	50.00	57.89	56.10
1 if Sellers	9.10	9.22	7.46	6.58	7.31
1 if Manufacturing	5.74	7.75	5.22	3.29	6.10
Hours work per week	34.19	35.24	37.41	34.57	37.16
Wages in Ghana Cedis	679.77	738.84	1005.06	1014.99	1227.36
1 if Permanent wage employee	72.92	78.05	82.84	84.87	88
					

Note: Individuals below 28 years and adults above 60 years are excluded. Disabled individuals are excluded. The figures for the dichotomous variables are in percentages.

## 6 Results

### 6.1 Private returns on education without controlling for selection and omitted variable bias

In this section, we provide the OLS results of the Mincerian earning function presented in equation (1). Table 4 provides the results of the regression estimates. Column (1) of the table does not include the education of father in the regression equation, but columns (2) and (3) include the education father in the regression equation. In column (2), we use years of education, while in column (3), we control for highest certificate of father. The authors prefer using the highest certificate of father This is because, unlike the human capital theory which is best explained by years of education, the signalling and social closure literature on the returns to education are better explained by highest level of certification than years of education. Thus, people of similar educational qualifications often obtain employment in similar environments and therefore build their friendships both in school and at the workplace. Notwithstanding our preference, we present results both for years of education of father and for highest certification of father.

**Table 4:** Returns to schooling without controlling for sample selection and OVB.

VARIABLES	Log wage	Log wage	Log wage
	(1)	(2)	(3)
Years of education	0.0582∗∗∗	0.0560∗∗∗	0.0560∗∗∗
	(0.0032)	(0.0033)	(0.0031)
1 if father have basic certificate			0.0418∗
			(0.0246)
1 if father have secondary certificate			0.1016∗
			(0.0527)
1 if father have diploma certificate			0.2156∗∗∗
			(0.0813)
1 if father have graduate or postgraduate			0.3237∗∗∗
			(0.0644)
Father's highest education level		0.0089∗∗∗	
		(0.0024)	
Observations	4533	4533	4533
R-squared	0.4196	0.4306	0.4246

Note: Although not reported in the table, all specification controls for gender, household head, literacy, religion, ethnic group, locality, hours worked, permanent employment, age, age squared, type of industry and duration of receiving payment were all used as covariates in the regression estimation. We also controlled for district fixed effects, which consist of 275 districts, and the standard errors in parentheses are clustered at the district level. The standard errors are bootstrapped with 100 replications. ∗ Means 10 percent significance level, ∗∗ is 5 percent significance level and ∗∗∗ is 1 percent significance level.

Notwithstanding the significant differences observed in Table 2, Table 3, the trend observed in both tables still suggests that one's father's educational level has some effect on wages. One major empirical issue is whether this observed association between the level of education of father and wages is due to the relationship between years of schooling and father's education or whether the level of certification of father has a direct influence on wages. Whether the level of certification of the father plays a role in determining the returns to education is a matter of empirical concern. Formal econometric modelling will be used to ascertain this research objective.

The results in Table 4 show that the inclusion of the education of the father slightly increases the explanatory power of the model. The R^2^ slightly increased from 41.96 % in the baseline estimate presented in column (1) to 43.06 (42.46) when years of education of father (highest certificate of father) are added to the baseline model. The baseline estimate in column (1) shows that the private returns to education in Ghana are 5.82 percent, and the figure slightly drops to 5.6 percent when the education of father is added to the regression equation. The results obtained here are quite similar to a recent study in Ghana by Ref. [33], who found the rate of return on education for women in Ghana to be 6.4 percent and that of men to be 5.3 percent. The results in columns (2) and (3) suggest the importance of controlling for father's education, as the coefficient of the education of father is significant even at the 1 percent confidence level. The results in column (3) also show that compared to the reference category—no certificate for father—as the level of certification of one's father increases, the wage of the individual likewise increases. The test of statistical significance indicates that the difference is significant. The significance of fathers' education in columns (2) and (3) and the strong evidence in the literature concerning the correlation of one's education and his father's education suggests that failing to control for father's education may lead to omitted variable bias.

Several empirical works on returns to education have shown that the error term in the Mincerian earning equation is likely to capture an unobserved variable such as ability, which is likely to affect both wages and years of schooling. For that matter, controlling for the education of the father without addressing the problem of endogeneity likely to be caused by ability would still make our regression estimate in Table 4 inconsistent. Apart from the omitted variable bias likely to be caused by the omission of the unobserved variable ability, people's decisions to participate in the labour force, especially women, are likely not to be random. Thus, failure to control for sample selection may also lead to biased estimates. In view of the above, the next two sections address both the selection and omitted variable bias problem using the two-stage estimation procedure explained in equation (2) through (5) in section 4.

### 6.2 Effect of spousal education on years of education

As explained in section (4), spouse education is used as an instrument for one's years of education. We therefore begin our instrumental variable analysis by first estimating the first-stage regression to see the partial correlation between spouse education and one's education. Since some members in our dataset were single at the time of the survey, there were some missing values for spouse education. The missing values for spouse education were almost 30 percent; therefore, dropping all those observations would significantly reduce our sample size. To maintain our sample size, we employed multiple imputations method to filling those missing values in spouse education.

Table 5 provides the regression results of the effect of spousal education on years of schooling. Similar to Table 4, column (1) serves as the baseline estimate, and columns (2) and (3) include the education of the father. The table shows that the instrument is positively related to years of education, and they are all significant at the 1 percent significance level. In column 1, an increase in the years of education of a spouse by one will increase years of schooling by 0.21. The partial effect of spouse education on years of schooling slightly decreases to 0.20 in both columns (2) and (3) when we control for the education of one's father. In all three estimations in Table (5), the Craig-Donald F-statistics are well above the conventional requirement of 10 [49]. Thus, our instrument—spouse education—is not weak.

**Table 5:** Effects of spousal education on total years of schooling.

VARIABLES	Years of education	Years of education	Years of education
	(1)	(2)	(3)
Years of spouse education	0.2125∗∗∗	0.2058∗∗∗	0.2070∗∗∗
	(0.0073)	(0.0079)	(0.0071)
1 if father have basic certificate			0.8924∗∗∗
			(0.0688)
1 if father have secondary certificate			1.7481∗∗∗
			(0.1471)
1 if father have diploma certificate			1.8497∗∗∗
			(0.1834)
1 if father have graduate or postgraduate			2.5399∗∗∗
			(0.2634)
Father's highest education level		0.1345∗∗∗	
		(0.0085)	
Craig Donald F-statistics	430.98	379.92	410.10
Observations	22,123	22,123	22,123
R-squared	0.6166	0.6382	0.6249

Note: Although not reported in the table, all specification controls for gender, household head, literacy, religion, ethnic group, locality, hours worked, permanent employment, age, age squared, type of industry and duration of receiving payment were all used as covariates in the regression estimation. We also controlled for district fixed effects, which consist of 275 districts, and the standard errors in parentheses are clustered at the district level. The standard errors are bootstrapped with 100 replications. ∗ Means 10 percent significance level, ∗∗ is 5 percent significance level and ∗∗∗ is 1 percent significance level.

### 6.3 The role of father in returns to schooling

Our interest in this section is to estimate the causal impact of years of schooling on wages. Thus far, we have demonstrated in section 6.1 that there is a correlation between years of schooling and wages, and section 6.2 principally discusses the first-stage regression of the IV method that we are about to consider in this section. In Table 6, we begin our analysis by estimating our baseline model that excludes the education of the father in column (1) and then controls for the education of the father in columns (2) and (3). This is done to check if the coefficient of the education of father in the IV estimation would be insignificant. Since the inverse Mills ratio (IMR) is estimated and added as a covariate, we used bootstrapped standard errors in the rest of the regression estimates.

**Table 6:** Returns to schooling controlling for both sample selection and OVB.

VARIABLES	Log wage	Log wage	Log wage
	(1)	(2)	(3)
Years of education	0.0594∗∗∗	0.0537∗∗∗	0.0561∗∗∗
	(0.0087)	(0.0091)	(0.0088)
1 if father have basic certificate			0.0413
			(0.0256)
1 if father have secondary certificate			0.1003∗
			(0.0543)
1 if father have diploma certificate			0.2158∗∗∗
			(0.0774)
1 if father have graduate or postgraduate			0.3233∗∗∗
			(0.0646)
Father's highest education level		0.0092∗∗∗	
		(0.0026)	
Inverse Mills Ratio	Yes	Yes	Yes
Observations	4533	4533	4533
R-squared	0.4028	0.4060	0.4061

Note: Although not reported in the table, all specification controls for gender, household head, literacy, religion, ethnic group, locality, hours worked, permanent employment, age, age squared, type of industry and duration of receiving payment were all used as covariates in the regression estimation. We also controlled for district fixed effects, which consist of 275 districts, and the standard errors in parentheses are clustered at the district level. The standard errors are bootstrapped with 100 replications. ∗ Means 10 percent significance level, ∗∗ is 5 percent significance level and ∗∗∗ is 1 percent significance level.

The results in column (1) show that an additional year of schooling is associated with an increase in wages by 5.94 percent. The results in columns (2) and (3) in Table 6 are slightly lower than the results in column (1). However, the confidence interval in the case of the IV estimation substantially overlapped. This is an indication that the instrument we used is able to minimise the omitted variable bias that may be caused by omitting the education of father. Even though the instrument may minimise the bias when father's education is not included in the IV estimation, the coefficient of years of schooling of father in column (2) and the dummies of levels of education in column (3) are still significant, suggesting that omitting fathers' education in the IV estimation is inappropriate. Findings from our study are similar to earlier papers that have found results from the IV regression to be slightly larger than OLS [33,39,50].

The regression results in Table 4, Table 6 provide solid evidence that the returns to education in Ghana also depend on the education of one's father. This observation can be tested statistically, creating five interaction terms—which is done by interacting one's years of education with different levels of certification of the education of the father. The levels of fathers' certification that interact with years of education are the dichotomous variables: (1) father has no certificate, (2) father has a basic certificate, (3) father has a secondary certificate, (4) father has a diploma certificate and (5) father has a bachelor's degree or higher certificate. Table 7 presents the result on how the education of fathers' level of certification influences returns to schooling.

**Table 7:** Returns to schooling and father's educational background.

VARIABLES	Father has No certificate	Father has Basic certificate	Father has Secondary certificate	Father has Diploma certificate	Father has Degree or higher
	(1)	(2)	(3)	(4)	(5)
Years of education	0.0637∗∗∗	0.0593∗∗∗	0.0585∗∗∗	0.0586∗∗∗	0.0571∗∗∗
	(0.0094)	(0.0090)	(0.0089)	(0.0088)	(0.0086)
Years of education∗No certificate	−0.0103∗∗∗				
	(0.0026)				
Education∗Basic certificate		−0.0000			
		(0.0023)			
Education∗Secondary certificate			0.0049		
			(0.0037)		
Education∗Diploma certificate				0.0113∗∗	
				(0.0052)	
Education∗Degree and above					0.0192∗∗∗
					(0.0039)
Linear combination of the interaction term and education	0.0535∗∗∗	0.0593∗∗∗	0.0634∗∗∗	0.0699∗∗∗	0.0763∗∗∗
	(0.0077)	(0.0077)	(0.0081)	(0.0099)	(0.0083)
Observations	4533	4533	4533	4533	4533
R-squared	0.4216	0.4188	0.4191	0.4197	0.4217

Note: Although not reported in the table, all specification controls for gender, household head, literacy, religion, ethnic group, locality, hours worked, permanent employment, age, age squared, type of industry and duration of receiving payment were all used as covariates in the regression estimation. We also controlled for district fixed effects, which consist of 275 districts, and the standard errors in parentheses are clustered at the district level. The standard errors are bootstrapped with 100 replications. The interaction term is years of schooling interacted with highest certification of father. ∗ Means 10 percent significance level, ∗∗ is 5 percent significance level and ∗∗∗ is 1 percent significance level.

Apart from the interaction between years of education and father having a basic certificate and years of education and father having a secondary certificate, which are not significant in Table 7, the rest of the interactions are significant. Interestingly, the coefficient of the interaction term begins at a higher negative value for the interaction between years of schooling and father having no certificate and then starts to increase until it reaches 1.92 percent for the interaction years of schooling and father having a bachelor's degree or higher. The results reveal that an additional year of schooling increases wages by 5.35 percent for individuals whose fathers do not have a certificate. The returns to schooling increase along the different levels of certification until they reach 7.63 for those individuals who have fathers who owned a first degree certificate or higher. It can simply be estimated from Table 7 that the returns to schooling for individuals whose fathers hold a bachelor's degree or higher are 2.3 percent higher than their counterparts whose fathers have no educational certificate. Using U.S. data [51], also had similar findings that the education of one's father has an effect on his/her wages.

It is possible that the effect of the education of the father on the returns to schooling may not be uniform across sectors. One can argue that the impact of the education of the father on returns to schooling may be stronger in sectors that have higher wages since parents with higher social capital (equivalently higher education) may lobby for their children to be employed in such high-paid jobs. In contrast, others may also argue that the competitiveness of higher-paid jobs may cause management to fill vacant positions with the best qualified individuals, and if that happens, then the education of the father may have little effect in sectors with higher-paid jobs.

Due to the above conflicting a priori theoretical prediction on how the education of fathers may shape the returns to schooling for different employment sectors that have significant wage differentials, we provide empirical analysis for two separate employment sectors. The two sectors considered are employment in the private or public sector and employment in the formal or informal sector. Table 8 presents the regression on how the education of fathers shapes returns to schooling for private and public sector employment.

**Table 8:** Returns to schooling in private/public institutions across different levels of father's educational certification.

VARIABLES	Father has No certificate	Father has Basic certificate	Father has Secondary certificate	Father has Diploma certificate	Father has Degree or higher
	(1)	(2)	(3)	(4)	(5)
.**Private Organisations**.					
Years of education	0.0618∗∗∗	0.0562∗∗∗	0.0548∗∗∗	0.0536∗∗∗	0.0522∗∗∗
	(0.0146)	(0.0128)	(0.0131)	(0.0130)	(0.0126)
Years of education∗No certificate	−0.0107∗∗				
	(0.0043)				
Education∗Basic certificate		−0.0055∗			
		(0.0030)			
Education∗Secondary certificate			0.0055		
			(0.0058)		
Education∗Diploma certificate				0.0297∗∗	
				(0.0115)	
Education∗Degree and above					0.0331∗∗∗
					(0.0071)
Linear combination of the interaction term and education	0.0512∗∗∗	0.0508∗∗∗	0.0603∗∗∗	0.0833∗∗∗	0.0853∗∗∗
	(0.0116)	(0.0111)	(0.0126)	(0.0139)	(0.0137)
Observations	3253	3253	3253	3253	3253
R-squared	0.4001	0.3991	0.3988	0.4019	0.4041
.**Public Organisations**.					
Years of education	0.0689∗∗∗	0.0675∗∗∗	0.0680∗∗∗	0.0678∗∗∗	0.0657∗∗∗
	(0.0178)	(0.0170)	(0.0175)	(0.0174)	(0.0186)
Years of education∗No certificate	−0.0054				
	(0.0041)				
Education∗Basic certificate		−0.0003			
		(0.0028)			
Education∗Secondary certificate			−0.0025		
			(0.0048)		
Education∗Diploma certificate				−0.0029	
				(0.0042)	
Education∗Degree and above					0.0034
					(0.0042)
Linear combination of the interaction term and education	0.0635∗∗∗	0.0672∗∗∗	0.0656∗∗∗	0.0649∗∗∗	0.0691∗∗∗
	(0.0149)	(0.0154)	(0.0162)	(0.0161)	(0.0160)
Observations	1280	1280	1280	1280	1240
R-squared	0.5853	0.5848	0.5846	0.5848	0.5861

Note: Although not reported in the table, all specification controls for gender, household head, literacy, religion, ethnic group, locality, hours worked, permanent employment, age, age squared, type of industry and duration of receiving payment were all used as covariates in the regression estimation. We also controlled for district fixed effects, which consist of 275 districts, and the standard errors in parentheses are clustered at the district level. The standard errors are bootstrapped with 100 replications. The interaction term is years of schooling interacted with highest certification of father. ∗ means 10 percent significance level, ∗∗ is 5 percent significance level and ∗∗∗ is 1 percent significance level.

We divided the sample into two—those working in the private sector and those working in the public sector. The total sample size for those working in the public sector is 1280 and that of those working in the private sector is 3253. Table 8 provides results on how the education of fathers shapes the private returns of schooling in both private and public sector employment in Ghana. Our interest in Table 8 is the significance of the interaction terms and the coefficient of the linear combination of the years of education and the interaction term.

It can clearly be seen from the table that all the interaction terms are not significant for those employed in the public sector. This suggests that the education of the father does not contribute to returns on schooling in the public sector. The table reveals that the returns to education for public sector employment are between 6.4 and 7.0 percent. In the case of private sector employment, we found that the education of the father shaped the returns to schooling in that sector.

The table reveals that people with their fathers having tertiary education (i.e., diploma, degree or higher) are more likely to obtain jobs in well-paid private sector institutions, and their returns are far higher than those in the public sector. In contrast, the returns to education for private sector employees whose parents do not have tertiary education are smaller than their counterparts who are likely to obtain employment in the public sector. Thus, our regression estimation suggests that individuals who have parents without tertiary education would greatly benefit if they get employment in the public sector rather than in the private sector. The evidence presented in Table 8 shows that private sector employment opportunities are more highly dependent on parental education than public sector employment.

Similar to the private‒public sector employment analysis, Table 9 provides an analysis for formal-informal employment. Our study used the same formal-informal employment construct in the dataset we are using. Similar to Table 8, our interest is in the interaction term and the linear combination of education and the interaction term. Apart from those with fathers who have a degree or higher certificate, the results in Table 9 reveal that the education of fathers does not have a significant effect on returns to schooling for informal sector employment. Similar to informal sector employment, only the interaction term between years of schooling and father having no certificate is significant at the 5 percent confidence level. The rest of the interaction terms are not significant at the 5 percent confidence level. Given the results obtained in Table 9, we conclude that the effect of the education of the father in shaping returns to schooling for informal sector employment is minimal. Similar things can be said about formal sector employment. An important observation from the table is that the returns to schooling for informal sector employment are 2–3.2 percent lower than those for formal sector employment, which affirms a study in Ghana by Ref. [52] that found that the labour market in Ghana has a majority of Ghanaian workers locked up in the lower tier of the informal economy, which has very low earnings compared to formal sector employment.

**Table 9:** Returns to schooling in formal/informal employment given different levels of father's educational certification.

VARIABLES	Father has No certificate	Father has Basic certificate	Father has Secondary certificate	Father has Diploma certificate	Father has Degree or higher
	(1)	(2)	(3)	(4)	(5)
.**Formal Employment**.					
Years of education	0.0758∗∗∗	0.0724∗∗∗	0.0737∗∗∗	0.0732∗∗∗	0.0698∗∗∗
	(0.0141)	(0.0134)	(0.0142)	(0.0141)	(0.0145)
Years of education∗No certificate	−0.0074∗∗				
	(0.0033)				
Education∗No certificate		−0.0055∗			
		(0.0028)			
Education∗Secondary certificate			0.0002		
			(0.0048)		
Education∗Diploma certificate				0.0046	
				(0.0067)	
Education∗Degree and above					0.0076∗
					(0.0042)
Linear combination of the interaction term and education	0.0684∗∗∗ (0.0130)	0.0669∗∗∗ (0.0123)	0.0739∗∗∗ (0.0132)	0.0778∗∗∗ (0.0130)	0.0774∗∗∗ (0.0132)
Observations	1521	1521	1521	1521	1521
R-squared	0.3822	0.3855	0.3813	0.3825	0.3875
.**Informal Employment**.					
Years of education	0.0394∗∗	0.0377∗∗	0.0368∗∗	0.0364∗∗	0.0368∗∗
	(0.0182)	(0.0151)	(0.0152)	(0.0152)	(0.0149)
Years of education∗No certificate	−0.0030				
	(0.0056)				
Education∗No certificate		−0.0021			
		(0.0035)			
Education∗Secondary certificate			0.0044		
			(0.0062)		
Education∗Diploma certificate				0.0155	
				(0.0115)	
Education∗Degree and above					0.0205∗∗
					(0.0084)
Linear combination of the interaction term and education	0.0364∗∗∗	0.0355∗∗∗	0.0413∗∗∗	0.0520∗∗∗	0.0573∗∗∗
	(0.0133)	(0.0131)	(0.0141)	(0.0152)	(0.0156)
Observations	3012	3012	3012	3012	3012
R-squared	0.3915	0.3914	0.3914	0.3919	0.3923

Note: Although not reported in the table, all specification controls for gender, household head, literacy, religion, ethnic group, locality, hours worked, permanent employment, age, age squared, type of industry and duration of receiving payment were all used as covariates in the regression estimation. We also controlled for district fixed effects, which consist of 275 districts, and the standard errors in parentheses are clustered at the district level. The standard errors are bootstrapped with 100 replications. The interaction term is years of schooling interacted with highest certification of father. ∗ means 10 percent significance level, ∗∗ is 5 percent significance level and ∗∗∗ is 1 percent significance level.

## 7 Robustness check

This section discusses the robustness of our results with respect to two major considerations. First, we cluster the standard errors by birthyear or by multistage clustering to see if the significance obtained in our regression results would change. In the final section, we try to use the double-header sample correction to see if the estimate for returns to schooling would significantly change. This robustness check is premised on the fact that two selection processes occur in the labour market. The first selection process happens as a result of nonrandom participation in the labour force, as discussed earlier. The second selection process is premised on the hiring decision of employers. Due to imperfect information, employers may use group characteristics to hire individuals, and this nonrandom selection into wage employment if not controlled for may also lead to a biased estimate [45,45,53] [54],.

### 7.1 Standard error clustering

To date, all the standard errors of the regression estimates provided in Table 4, Table 5, Table 6, Table 7, Table 8, Table 9 have been clustered at the district level. In this section, we provide results for estimations that cluster the standard errors at the year of birth and then also present results that use the multistage clustering method by Ref. [55]. In the case of multistage standard error clustering, we used clustering at both district and year of birth. Table 10, Table 11 present the results for clustering at the birth year and that of multistage clustering, respectively. Comparing the significance of Table 10, Table 11 with that of Table 6, there is no difference between the significance obtained in Table 6 and that obtained in Table 10, Table 11. Although the type of clustering changes the standard errors, the change in standard errors due to the type of clustering is not large enough to affect the significance. The results presented in both Table 10, Table 11 show that our regression estimate is highly robust to standard error clustering.

**Table 10:** Returns to schooling (clustering standard errors at year of birth).

VARIABLES	Log wage	Log wage	Log wage
	(1)	(2)	(3)
Years of education	0.0594∗∗∗	0.0537∗∗∗	0.0561∗∗∗
	(0.0080)	(0.0081)	(0.0080)
1 if father have basic certificate			0.0413∗∗
			(0.0200)
1 if father have secondary certificate			0.1003∗
			(0.0565)
1 if father have diploma certificate			0.2158∗∗∗
			(0.0804)
1 if father have graduate or postgraduate			0.3233∗∗∗
			(0.0663)
Father's highest education level		0.0092∗∗∗	
		(0.0029)	
Inverse Mills Ratio	Yes	Yes	Yes
Observations	4533	4533	4533
R-squared	0.4186	0.4289	0.4231

Note: Although not reported in the table, all specification controls for gender, household head, literacy, religion, ethnic group, locality, hours worked, permanent employment, age, age squared, type of industry and duration of receiving payment were all used as covariates in the regression estimation. We also controlled for district fixed effects, which consist of 275 districts, and the standard errors in parentheses are clustered at year of birth. The standard errors are bootstrapped with 100 replications. ∗ Means 10 percent significance level, ∗∗ is 5 percent significance level and ∗∗∗ is 1 percent significance level.

**Table 11:** Returns to schooling (multistage clustering of standard errors).

VARIABLES	Log wage	Log wage	Log wage
	(1)	(2)	(3)
Years of education	0.0594∗∗∗	0.0537∗∗∗	0.0561∗∗∗
	(0.0086)	(0.0091)	(0.0088)
1 if father have basic certificate			0.0413∗
			(0.0237)
1 if father have secondary certificate			0.1003∗
			(0.0590)
1 if father have diploma certificate			0.2158∗∗∗
			(0.0735)
1 if father have graduate or postgraduate			0.3233∗∗∗
			(0.0628)
Father's highest education level		0.0092∗∗∗	
		(0.0029)	
Inverse Mills Ratio	Yes	Yes	Yes
Observations	4533	4533	4533
R-squared	0.4186	0.4289	0.4231

Note: Although not reported in the table, all specification controls for gender, household head, literacy, religion, ethnic group, locality, hours worked, permanent employment, age, age squared, type of industry and duration of receiving payment were all used as covariates in the regression estimation. We also controlled for district fixed effects, which consist of 275 districts, and the standard errors in parentheses are clustered at year of birth and district. The standard errors are bootstrapped with 100 replications. ∗ Means 10 percent significance level, ∗∗ is 5 percent significance level and ∗∗∗ is 1 percent significance level.

### 7.2 Controlling for double-header sample selection

As a further check of the robustness of our results, we assumed that there exists a possibility of double-header sample selection instead of the single sample selection assumed in Table 6. The purpose of this robustness check is that some earlier studies have argued that the wage of a worker is only observed when the worker is employed and that the only way people obtain wage employment is first to decide to participate in the labor market, and then second is the decision of employers to hire that worker. Apart from the decision of employers in hiring a worker, some workers (especially women) self-select themselves to self-employment due to its flexibility when compared to wage employment [56,53]. In view of the above, some earlier empirical researchers have argued that the.

Mincerian earning equation follows a double-header selection process, and therefore, a consistent estimate can only be obtained when one considers both selection into labor force participation and that of employment [53,54]. If the data generating mechanism in Ghana follows double-header selection, then the single sample selection used in our estimation may provide a biased result. Therefore, we estimate the wage equation using the double-header correction procedure proposed in Ref. [57].

Table 12 provides results for the double-header correction procedure that assumes that the error terms in the two selection equations do not correlate. In this case, it is assumed that individuals make the decision to participate, after which they are then selected into wage employment or self-employment. In estimating the IMR in Table 12, we follow [58], which requires using two separate probit regressions for the participation equation and the wage employment equation, after which the inverse Mills ratios are calculated from each of the estimations. Clearly, the inverse Mills ratios in Table 12 suggest that there is only one selection that is significant; therefore, our baseline estimates that correct for single selection are most likely not biased. The private returns to schooling in Table 12 are slightly lower than those in Table 6, but the confidence intervals largely overlap each other, suggesting that the single correction is sufficient. For example, while the returns to education in Table 12 column (1) are 5.75 percent, we found them to be 5.94 in Table 6 column (1). The confidence interval for Table 12 column (1) is (0.03397,0.0666), while that of Table 6 column (1) is (0.0423,0.0764), which shows a significant overlap in confidence intervals, indicating that using the single correction method is likely to give consistent estimates.

**Table 12:** Returns to schooling controlling for double selection with no correlation in errors.

VARIABLES	Log wage	Log wage	Log wage
	(1)	(2)	(3)
Years of education	0.0575∗∗∗	0.0519∗∗∗	0.0540∗∗∗
	(0.0091)	(0.0093)	(0.0092)
1 if father have basic certificate			0.0413
			(0.0257)
1 if father have secondary certificate			0.1011∗
			(0.0542)
1 if father have diploma certificate			0.2192∗∗∗
			(0.0778)
1 if father have graduate or postgraduate			0.3232∗∗∗
			(0.0642)
Father's highest education level		0.0093∗∗∗	
		(0.0026)	
Inverse Mills Ratio 1	−0.0632	−0.0421	−0.0646
	(0.1651)	(0.1681)	(0.1621)
Inverse Mills Ratio 2	−0.0705∗∗	−0.0721∗∗	−0.0739∗∗
	(0.0348)	(0.0350)	(0.0345)
Observations	4533	4533	4533
R-squared	0.4193	0.4295	0.4238

Note: Although not reported in the table, all specification controls for gender, household head, literacy, religion, ethnic group, locality, hours worked, permanent employment, age, age squared, type of industry and duration of receiving payment were all used as covariates in the regression estimation. We also controlled for district fixed effects, which consist of 275 districts, and the standard errors in parentheses are clustered at the district level. The standard errors are bootstrapped with 100 replications. ∗ Means 10 percent significance level, ∗∗ is 5 percent significance level and ∗∗∗ is 1 percent significance level.

Table 13 provides results for the double-header correction procedure that assumes that the error terms of the two selection equations correlate. In this case, the decision to participate in the labour force and to work in wage employment are taken simultaneously. To estimate the two inverse Mills ratios when the error terms correlate, we follow the approach suggested by Ref. [57], which assumes that the wage equation and the two selection equations follow a trivariate normal distribution. Again, the inverse Mills ratio in Table 3 suggests that correcting for single selection in the baseline model (Table 6) is not out of place. Similar to that of Table 12, the private returns to schooling in Table 13 are slightly lower than that of Table 6, but the confidence intervals largely overlap each other, suggesting that the single correction is sufficient. The results indicate that using the single correction method is likely to give consistent estimates.

**Table 13:** Returns to schooling controlling for double selection with correlation in errors.

VARIABLES	Log wage	Log wage	Log wage
	(1)	(2)	(3)
Years of education	0.0584∗∗∗	0.0525∗∗∗	0.0548∗∗∗
	(0.0088)	(0.0091)	(0.0089)
1 if father have basic certificate			0.0424∗
			(0.0256)
1 if father have secondary certificate			0.1027∗
			(0.0530)
1 if father have diploma certificate			0.2252∗∗∗
			(0.0771)
1 if father have graduate or postgraduate			0.3194∗∗∗
			(0.0640)
Father's highest education level		0.0095∗∗∗	
		(0.0026)	
Lamda 1	0.1152	0.0603	0.1252
	(0.0923)	(0.0871)	(0.0935)
Lamda 2	0.3633∗∗∗	0.3853∗∗∗	0.3565∗∗∗
	(0.0969)	(0.0995)	(0.0948)
Observations	4533	4533	4533
R-squared	0.4218	0.4326	0.4268

Note: Although not reported in the table, all specification controls for gender, household head, literacy, religion, ethnic group, locality, hours worked, permanent employment, age, age squared, type of industry and duration of receiving payment were all used as covariates in the regression estimation. We also controlled for district fixed effects, which consist of 275 districts, and the standard errors in parentheses are clustered at the district level. The standard errors are bootstrapped with 100 replications. ∗ means 10 percent significance level, ∗∗ is 5 percent significance level and ∗∗∗ is 1 percent significance level.

## 8 Discussion of findings

In this study, we investigate how the education of a father shapes the returns to schooling. Spouse education is used as an instrument to address the possibility of omitted variable bias due to the unobserved variable ability, which could not be directly controlled in our model. Our extensive review of the literature shows that all empirical studies on returns to schooling have found a direct relationship between years of schooling and wages [31]. reported that the returns to schooling in Ghana are between 5 and 7 percent, and a recent study by Ref. [33] also affirms the findings made by Ref. [31]. After controlling for all the possible sources of endogeneity, our study found the average returns to schooling in Ghana to be 5.4–6.0 percent, and this figure is far below the world average of 9.7 percent and 12.4 percent for sub-Sahara Africa based on estimates of [59]. This shows that the returns to schooling in Ghana are quite low compared to other countries in the world or in sub-Sahara Africa.

There could be several reasons for this lower return to schooling in the country. One such factor may be the quality of Ghanaian educational systems compared to the rest of the world. Some earlier studies have empirically and theoretically shown that the quality of education is likely to affect the returns to schooling [27,28]. After all, common sense will tell us that human capital accumulation is principally acquired through the skills and knowledge that the educational system offers but not the years spent in school. If there is less human capital accumulation in the Ghanaian educational sector, then there is more likelihood that the returns on schooling in the country would be lower. There is no doubt that the standard of the educational system in Ghana is low. A clear reason to believe that Ghanaian educational standards are low compared to the rest of the world is Ghana's performance on the Trends in International Mathematics and Science Study (TIMSS) in 2011 when it last participated. Out of the 46 countries that participated in the TIMSS assessment in 2011, Ghana placed 45th in mathematics and 46th in science. The country's performance in earlier participation is no different from what pertained in 2011 before it exited from the assessment.

The second possible reason explaining the low returns to schooling in the country may be the centralised nature of educational decisions, which is often determined by the political class in the country without much consultation from the key players in the educational sector within the country. For example, in 2007, the ruling government at the time changed the years of secondary education to 4 years only to be reverted to 3 years in 2009 after the ruling government lost power to the opposition party. In the 2021/2022 academic year, the ruling government changed the content and structure of pre-secondary education in the country without much consultation with key stakeholders. The over politicisation of the Ghanaian educational system has weakened the power of technocrats entrusted to managing the sector. Teachers, head teachers, and educational administrators have little power to tailor Ghanaian education towards the needs of students, as the political class has taken over the sector and is using the system for political gains. Additionally, the educational system in the country places much emphasis on remote memorisation, and little room is allowed for student engagement in the classroom. For example, on 17 June 2021, the minister of education Dr. Yaw Osei Adutwum said they are developing a curricular that would eliminate the “chew-and-pour”/rote learning attitude of Ghanaian students and emphasised that the new curricular would provide graduates that possess the necessary soft skills needed to make them useful in the labour market [60].

Finally, corruption in Ghana could also be playing a part in the low returns to schooling in the country. Hiring employees through nepotism and corruption may prevent more educated and productive individuals from finding suitable jobs and, as a result, may disincentivise people from studying hard and improve their human capital. If promotion in the workplace is based on nepotism, corruption and favoritism instead of productivity, then there is a higher possibility that such practices will affect human capital accumulation. In a working environment where promotion is done on the basis of nepotism and favoritism, people prefer to create connections with management and people of higher authority rather than improving themselves to become more productive, which has high potential to reduce the returns to schooling. For example, a study in Ghana by Ref. [61] shows that military job matches through referrals in the British colonial era in the country did not result in recruiting more efficient people who were fit for the task. A recent study by Ref. [62] reveals that a higher proportion of formal wage employees received their first appointment through social networks rather than through formal hiring procedures. In view of the above, people would prefer to use any means in getting certificates without much effort in building their human capital, and this will likely reduce the returns to schooling, as people might spend more years in education but would be less productive.

Aside from the low returns to schooling observed in this study, we also found the returns to schooling to be dependent on the level of certification of one's father. The study reveals that one additional year of schooling increases wages by 5.3–7.6 percent depending on the level of certification of one's father. The study reveals that the return on schooling for people who have fathers who hold a bachelor's degree or higher is 2.3 percent higher than for those whose father does not have a certificate. The 2.3 percent difference alone is close to 40 % of the returns of those whose fathers do not have a certificate, indicating a strong influence of fathers' education on one's wages. Our finding is similar to a study in Brazil that investigated the effect of omitting family background on returns to schooling, which found the returns to schooling to fall by one-third when parental education was added to the wage equation [29]. The question then is how the education of a father affects the returns to schooling by an individual. We offer three possible reasons that are likely to explain this finding.

Our first argument hinges on knowledge transfer. To explain how knowledge transfer works, parents often either through direct or indirect means, supplement the formal education process of their children, and parents with a higher level of education would be able to do that better than their counterparts with a lower educational level. It has been noted that higher educated individuals spend more time with their children and therefore become more concerned about their school choice [63]. Educated parents may be more concerned about the brain development of their children and may therefore provide more nutritious diets to their children [64]. In view of the above, there is the likelihood that the education of someone with a father with a higher level of schooling is likely to be better than that of someone with a father with a lower educational level, even if they both have the same years of schooling or the same level of formal qualification. These differences may translate into their work output at the initial stages of their employment after school, thereby creating the observed wage differences found in our study.

The second possible mechanism through which father's education may affect one's returns to schooling is through social network. Parents with higher level of schooling are likely to have friends and class mates who take decisions at both industrial level and economy at large. For this reason, they may be able to use their social network to aid their children to secure employment in lucrative institutions immediately after they graduate from school.

Finally, children with parents with higher education often have their parents already working in lucrative occupations and this reason is likely to explain the potential influence that educated parents may have on the labour market. Parents with a higher level of education are most likely to be well connected in society, as they themselves or might have friends that are placed in more influential positions in the labour market. Thus, educated parents are able to secure well-paid jobs for their children. In a labour market where job vacancies are relatively scarce compared to individuals searching for jobs, the duration of employment is more likely to be shorter for children who have parents with higher education, as such parents would use their connections to help their children secure jobs. Thus, the short duration of unemployment and the possibility of obtaining jobs in well-paid occupations are some of the possible reasons accounting for the higher returns of education of children having educated fathers.

Our findings suggest that the education of fathers does not matter in determining the returns to schooling for public sector employment but does matter for private sector employment. In the case of formal-informal employment sectors, we found some marginal evidence on the contribution of the education of fathers shaping the returns to schooling. Another important observation from our estimates suggests that the returns to schooling in the informal sectors of the economy are significantly lower when compared to those employed in the formal sector. This insight is similar to that of [30], who found the returns to schooling to be lower in the informal sector than in the formal sector. For the case of comparing private and public sector workers, our results are similar to Ref. [25] evidence from 28 European and Central Asian countries, which shows returns to schooling to be higher for people with tertiary certificates. The results from our study suggest that those with fathers who have university education have the advantage of obtaining employment offers from highly paid employment opportunities in the country, which is often found in the private sector.

It is a stylised fact that the private sector of Ghana's economy is more competitive and efficient than the public sector. In that sense, it is expected that the returns to schooling would be higher in the private sector than in the public sector, which is noncompetitive. For example [65], posits that the objectives of the private sector may be different from those of the public sector. It is assumed that the public sector may pay less skilled workers higher wages than their productivity, and this is unlikely to happen in the more competitive private sector. However, for political reasons, managers of the public sector may be unwilling to pay high-skilled workers the necessary premium not to leave to the private sector. In view of the above, two deductions can be made from our findings. First, employers in the private sector would like to put up measures that would attract efficient workers to make them more competitive, and one of the ways they may adopt is to depend on referrals from current or old employees since there is information asymmetry in the labour hiring process. Thus, parents with higher education are more likely to help their children, as they may have the required networks that would help them secure those jobs. The second probable explanation for why parental education has more influence in the private sector than in the public sector may be due to nepotism, as higher educated parents may likely occupy top managerial positions of those highly paid jobs and therefore have influence on the hiring of workers in the organisation. Given that the private sector is a competitive sector, relying on referrals to determine worker productivity is more likely to be the case. However, whether nepotism or referrals minimise information asymmetry is a question empirical test, and the dataset we are using is insufficient to address such empirical questions.

## 9 Conclusion

This study investigates the causal link between education and wages by using spousal education as an instrument for years of schooling. The evidence presented in the study demonstrates the importance of controlling for the education of one's father in returns to schooling. The findings from the study are consistent with other empirical studies that have found a direct causal relationship between years of schooling and wages. The study revealed that returns to education in the country are in the range of 5.4–6 percent.

A further revelation from the study reveals that one additional year of schooling increases wages by 5.3–7.6 percent depending on the level of certification of one's father. The study reveals that the return on schooling for people who have fathers who hold a bachelor's degree or higher is 2.3 percent higher than their counterparts whose father does not have a certificate. However, the results suggest that the level of education of fathers does not matter for public sector workers. In contrast, we found father's education to have an effect on the returns of education in the private sector. The paper also compared the returns to schooling between formal and informal sector employment, and our results reveal some marginal evidence of fathers' education shaping the returns to schooling in both sectors. An important observation from our estimates suggests that the returns to schooling in the informal sectors of the economy are significantly lower when compared to the formal sector.

In all, the study set out to find the returns to education in Ghana and examine how father's education shape returns to education in the country. Our findings indicate positive returns to education in the country. The study further reveals that the higher the education of one's father, the bigger the returns to schooling. The study further sought out to investigate whether heterogeneity exist in the returns to schooling in regard to private/public or formal/informal sectors of the economy and how fathers education shape these heterogeneities. The study found some evidence of father's education shaping the returns of education in both the formal and informal sectors of the economy. The study also found father's education to play a role in the returns to education with regard to the private sector but there was no such evidence with regard to the public sector.

The realisation of the significant role of parent education on children's educational outcomes presents two interesting issues. First, from a policy perspective, since parents seem to have influence in securing jobs for their children, it is important to institute structures that would reduce the extent to which parents can influence job acquisition for their children. When job selection is done in an open manner such that whoever deserves it gets the job, the returns to education may increase. To achieve this, the state should enact policies that may reduce information asymmetries so that employers may have better information about prospective employees during the selection process of prospective employees. In that case, referrals may not be important since adverse selection may be significantly reduced.

Second, the parental effect on children's return on education implies that enhancing the educational attainment of the current generation may yield positive outcomes for future generations individually and as a nation. It is in this light that policymakers need to ensure educational policies in the country promote retention at the basic to senior high school levels and make higher education accessible and affordable to enable many to enroll. Current educational conditions in the country leave much to be desired. The earlier authorities paid attention to the shortfalls in the Free Compusory Universal Basic Education policy, Free SHS policy, funding option for tertiary students and infrastructural deficits at all levels of education in the country, the better it would be.

The study also found returns to schooling to be higher in the formal sectors of the economy; therefore, we recommend that the state implement policies that would bring much of the labour force into formal employment. An observation of the trend of school enrolment and completion in the country is increasing, suggesting that parents seem to be aware of the potential benefits of investing in their children's education. This appears to hold true even for uneducated parents, who are apparently compensating for their own lack of education by educating their children. This means that policies that are designed to reduce the burden of poor households in caring for their children's education may increase school enrolment and attainment, and this would in the long run lead to upwards social mobility, which would in effect bring about a reduction in income inequalities.

Ghana like many developing countries share similar educational and labour market characteristics. Several developing countries like Ghana have poor educational infrastructures and has high student to teacher ratio. The labour market developing countries are often characterized by high informality with very few of the active adult population engaged in wage employment. Given the similarities in both education and labour market outcomes among developing countries, the findings from this paper is likely to be relevant to other developing countries, especially those countries in sub-Sahara Africa which share similar educational and labour market characterstics and have similar social dynamics like that of Ghana.

There are few limitations of the study. The first limitations of the study border the instrument used for the study. Approximately 30 percent of the total observations of education of spouse were missing and therefore we have to rely on multiple imputations to compute the education of spouses for those that were missing. Dropping those observations without spouse education would have also reduced the sample size significantly. If the predictions of spouse education through multiple imputation are inaccurate due to the sheer size of the missing values of spouse education, then our estimations may not reflect the actual returns of education in the country. The second limitation of the study is the concentration on wage employees. Nonwage employees form the larger share of the labour force in the country, and therefore, understanding the returns to schooling on that part of the labour market would have a wider policy implication. This limitation can be addressed by future studies that may have information on the earnings of nonwage workers so that the returns to schooling for self-employed workers can also be measured. It is possible that the returns to education may also depend on the type of programme studied, and future studies may need to evaluate whether the private returns to schooling depend on the type of programme offered in secondary school or at the tertiary level. Future studies can consider the effect of nepotism and referrals on wage differentials to broaden the discussion on the subject matter. The paper opens avenues for future research in terms of exploring other determinants that mediate the relationship between parental education and an individual's outcomes. Future studies can therefore extensively look at how access to information, social network and labour market opportunities mediate the relationship between parental education and one's returns to schooling.

## Data availability

The data used in the article is publicly available at the Website of Ghana Statistical Service and can be accessed using the link https://www2.statsghana.gov.gh/nada/index.php/catalog/97.

## CRediT authorship contribution statement

**Emmanuel AduBoahen:** Writing – original draft, Software, Methodology, Conceptualization. **Paul Hammond:** Writing – review & editing, Investigation. **Paul A. Kwakwa:** Writing – review & editing, Formal analysis, Conceptualization.

## Declaration of competing interest

The authors declare that they have no known competing financial interests or personal relationships that could have appeared to influence the work reported in this paper.

## Footnotes

1 Only few wage employees may be awarded a contract beyond age 60 years. Thus, including retirees will rather biased our estimates since the distribution of people with contract after age 60 years is likely not to be random.

2 Only 455 out of 31,305 (i.e. 1.45 %) of the total sampled observation are disabled and for wage employment only 38 out of 4533 (i.e. 0.8 %). It is unlikely for disability within the population to be systematic and therefore dropping these observations are unlikely to biased the regression estimates.
